# Prevalence of different tick species on livestock and associated equines and canine from different agro-ecological zones of Pakistan

**DOI:** 10.3389/fvets.2022.1089999

**Published:** 2023-01-06

**Authors:** Nazeer Hussain, Rana Muhammad Kamran Shabbir, Haroon Ahmed, Muhammad Sohail Afzal, Shafi Ullah, Abid Ali, Shumaila Irum, Syed Kamran-ul-Hassan Naqvi, Jianhai Yin, Jianping Cao

**Affiliations:** ^1^Department of Biosciences, COMSATS University Islamabad, Islamabad, Pakistan; ^2^Department of Zoology, Division of Science and Technology, University of Education, Lahore, Pakistan; ^3^Department of Life Sciences, School of Science, University of Management and Technology, Lahore, Pakistan; ^4^Department of Zoology, Abdul Wali Khan University Mardan, Mardan, Khyber Pakhtunkhwa, Pakistan; ^5^Department of Zoology, University of Gujrat, Gujrat, Pakistan; ^6^National Institute of Parasitic Diseases, Chinese Center for Disease Control and Prevention (Chinese Center for Tropical Diseases Research), Shanghai, China; ^7^Key Laboratory of Parasite and Vector Biology, National Health Commission of the People's Republic of China, Shanghai, China; ^8^World Health Organization Collaborating Center for Tropical Diseases, Shanghai, China; ^9^The School of Global Health, Chinese Center for Tropical Diseases Research, Shanghai Jiao Tong University School of Medicine, Shanghai, China

**Keywords:** livestock, ticks, tick-borne diseases, agro-ecological zones, Pakistan

## Abstract

Ticks are ectoparasites that act as vectors for transmission of various pathogens to wild and domesticated animals and pose a serious threat to human health. Because of the hot and humid conditions in different agro-ecological zones of Pakistan, ticks are abundant and parasitize a variety of animals. The aim of this study was to identify different tick species and distribution on different hosts especially livestock, such as sheep, goat, cattle, buffalo, and camel, and livestock associated canines and equines, such as horse, donkey, and dog, across different agro-ecological zones of Pakistan. The ticks samples were collected and morphologically identified at genus and species level using morphological keys under stereomicroscope. A total of 2,846 animals were examined for the tick infestation, and 408 animals were tick-infested. Eleven tick species belonging to 4 genera were identified: *Hyalomma anatolicum, Hyalomma scupense, Hyalomma dromedarii, Hyalomma isaaci, Rhipicephalus microplus, Rhipicephalus haemaphysaloides, Rhipicephalus turanicus, Haemaphysalis cornupunctata, Haemaphysalis montgomeryi, Haemaphysalis bispinosa*, and *Ixodes kashmiricus*. The overall tick prevalence was 14.3%; host-wise infestation rate was 12.2% in sheep; 12.6%, goat; 11.7%, buffalo; 11.7%, cattle; 19.6%, camel; 27.4%, donkey; 23.5%, horse; and 24.3%, dog. Tick infestation of different animals differed on the basis of the zones. Camels showed the highest tick infestation rate in zones 1 and 2 (21.4 and 26.7%, respectively), whereas donkeys showed the highest infestation rate in zones 3, 4, 6, and 7 (25, 39.3, 3.3, and 21.4%, respectively). The infestation rates of *Hyalomma* and *Rhipicephalus* were the highest in zone 2 (71.4 and 52.9%, respectively). The infestation rate of *Hyalomma* was the highest (47.4%) in sheep; *Haemaphysalis* (46.9%), goat; *Rhipicephalus* (69.7%), buffalo; *Rhipicephalus* (62.3%), cattle; *Hyalomma* (70%), camel; *Ixodes* (60.9%), donkey; *Ixodes* (75%), horse; and *Rhipicephalus* (61.1%), dog. This study showed the diversity and infestation rate of different ticks with respect to their hosts and agro-ecological zones of Pakistan. High tick burdens and infestation rates are responsible for the spread of different tick-borne infections, resulting in loss of animal productivity and posing a threat to animal and human health. Understanding different tick species and their distribution across different zones will be helpful for developing efficient control strategies against different tick born infections.

## 1. Introduction

Ticks are the most important vectors for animal and human diseases worldwide, and these arthropod vectors are associated with different pathogenic microorganisms (1). The number of tick-borne pathogens is increasing with the passage of time because of the discovery of novel species of bacteria, viral pathogens, and protozoan parasites (2). A specific association exists between vector-borne pathogens and their associated hosts, so it is very important to identify and characterize tick species. Because of the probable threat of ticks to humans and animals, further studies are needed to understand the relationship between tick-borne pathogens (TBP) and tick species (3).

Almost 80% of the world cattle population is affected by ticks and tick born diseases and India, Pakistan, and Bangladesh are included in the endemic regions. Several factors, including geo-climatic conditions, different animal species, understanding/knowledge of the farmers, and farm management practices, are responsible for the variations in tick prevalence in specific areas. A previous study has shown that different animal species have different tick populations (4).

Domesticated animals in tropical and subtropical areas like Pakistan are infected with tick-borne bacterial and parasitic diseases. Ticks as vectors and related tick-borne infections are prevalent in arid areas of Punjab, Pakistan (5). In a survey of ixodid ticks in Turkey, 11 tick species were isolated (6). *Rhipicephalus* ticks of cattle origin have a negative impact on livestock production, especially in tropical and subtropical areas. Four different *Rhipicephalus* tick species have been identified in different areas of Iran (7).

A recent study conducted on equines in Pakistan, where 5 species of hard ticks belonging to 3 genera were reported, these includes *Hyalomma, Rhipicephalus*, and *Haemaphysalis* (8). Similarly, *Hyalomma anatolicum* was found infesting horses in Balochistan, Pakistan (9), while hard tick *Nosomma monstrosum* was reported for the first time on Asian water buffalo in Pakistan (10). *Rhipicephalus sanguineus, Rhipicephalus turanicus*, and *Rhipicephalus haemaphysaloides* were detected for the first time infesting wild animals in Pakistan (11). Hard ticks of the genus *Hyalomma* can act as a vector for *Babesia* and *Theileria* species in most endemic parts of Asia and Africa (12, 13). Seeking the importance of soft ticks in Pakistan, previously a study was conducted revealing the life cycle of *Argas persicus* for the first time (14). In Pakistan, ticks have a wide distribution depending on ecological and geographical patterns (15). Among domesticated animals, dogs have been reported as risk factors for ticks and tick-borne infections (16). In a study performed at a cow farm in Rajanpur, Pakistan, the total economic loss due to ticks and associated tick-borne diseases in terms of decreased milk production, mortality, and medication was 13.83% (17). The livestock industry is seriously affected by tick-borne pathogens. A study conducted in Southern Punjab, Pakistan, to determine the prevalence of tick-associated diseases showed that parasite prevalence ranged from 10 to 41% depending upon geographical distribution (18). *Rhipicephalus* and *Hyalomma* ticks are the most common tick species prevalent in cows and buffaloes in Pakistan (19–21). According to a recent study conducted in different zones of KP (Khyber Pakhtunkhwa), Pakistan, 3 major tick species, *Rhipicephalus microplus, Rhipicephalus haemaphysaloides*, and *Rhipicephalus turanicus*, showed positive results for *Anaplasma marginale*, the causative agent of anaplasmosis (22). In a recent paper, future concerns about ticks and tick-borne diseases were discussed (23).

Therefore, the major objective of the present study was to assess tick infestation in livestock (cattle, buffalo, sheep, goats, and camels) and associated animals (donkey, horse, and dog) in different agro-ecological zones of Pakistan The findings of current study will help to produce a geographical correlation between ticks of different regions that infect a variety of animal hosts.

## 2. Materials and methods

### 2.1. Study area

**Figure 1:**
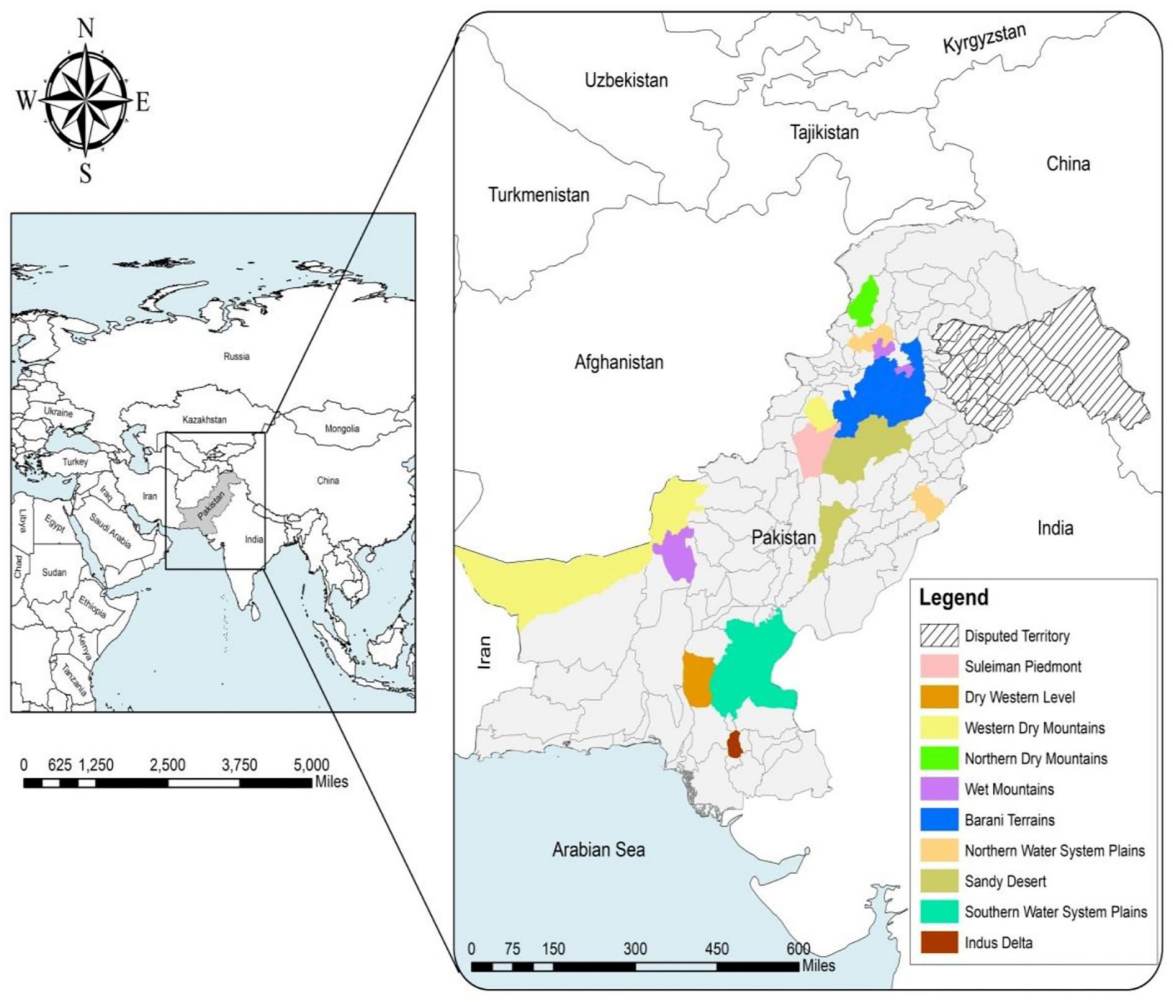
Map of different agro-ecological zones from which the ticks were collected for this study.

This current research work is conducted in different agro-ecological zones across Pakistan.^1^ The sampling duration was from June 2020 to September 2021. The study locations are depicted in Figure 1. Pakistan is largely a rural and agro-based country, so the major livelihood of the rural community is animal husbandry.^2^ In 2020, the contribution of the livestock industry was 60.6% to overall agriculture and 11.7% to the GDP (24). In 2020, there were ~41.2 million buffaloes, 49.6 million cattle, 5.4 million donkeys, 78.2 million goats, 30.9 million sheep, 1.1 million camels, and 0.4 million horses in Pakistan (24). The dog population is not reported officially in any set pattern, but about 15 million street dogs have been estimated in Pakistan.^3^^,^^4^ These animals are a potential source of ticks and tick-borne diseases. The climatic conditions in Pakistan are very diverse. The annual precipitation ranges between 50 and 3,000 mm. The average temperature is 40°C (see text footnote 1). The hot and humid climate greatly contributes to the growth and infestation rate of tick species on different animals. Moreover, precipitation plays a major role in the increased density of some tick species; hot and dry weather has been associated with a reduction in the density of ticks (25).

#### 2.1.1. Agro-ecological zone of Pakistan

The northern irrigated zone comprises, Multan, Vehari, Sahiwal, Lahore, Kasur, Faisalabad, Jhang, Sheikhupura, Gujranwala and parts of Bahawalnagar, districts of Rahim Yar Khan, Muzaffargarh, Sargodha, and Gujrat. Additionally, this zone includes the Peshawar and Mardan districts.

#### 2.1.2. Physiographic and climatic characteristics

Areas between Sutlej and Jhelum rivers; different flood plains and bar upland; Climate semi-arid to arid (east to southwest) subtropical continental; maximum (summer) and minimum (winter) temperatures are respectively 39.5°C and 6.2°C in the east and 41°C in the south west; mean annual rainfall is 300–500 mm in the east and 200–300 mm in the south west. Plains of Mardan and Peshawar's alluvial valleys Semi-arid subtropical continental climate; typical daily maximum (summer) and minimum (winter) temperatures are 43–44°C and 5°C, respectively; mean monthly rainfall ranges from 20–32 mm in both winter and summer.

#### 2.1.3. Soils and land use

Sand-clay loam soil with calcareous silt loams in the southern and central parts and around 15% saline-sodic soil in localized locations; loam and clay in the northern section. Crops grown *via* canal irrigation include citrus and mangoes in the central and southern regions, along with wheat, rice, sugarcane, oilseeds, and millets in the north. 5. Barani areas included in this zone are parts of D.I. Khan, Banu, Mianwali, Abbottabad, Rawalpindi, Gujrat, Gujranwala, Attock, Jhelum and Sialkot are included in this zone.

### 2.2. Sample size, tick collection, and preservation

A total of 26 districts were selected from all 10 agro-ecological zones of Pakistan depending upon precipitation and temperature variations. Random selection was performed in different villages depending on the premise that at least 3 host species exist in the sampled herd. The sampled animals were inspected for ticks by using a standardized protocol^5^; they were inspected thoroughly for the existence of tick infestation by physical examination and palpation. Ticks were sampled from different body sites of the animals, namely, ears, back of the neck, mammary glands, perineum, and under the tail base. The ticks were manually removed with forceps and immediately stored in safe-lock Eppendorf tubes containing 100% ethanol. The ticks were removed carefully from the host body to avoid damage to any tick part.

### 2.3. Morphological identification of tick specimens

A stereo microscope (SZ 61; Olympus, Japan) was used for the morphological examination of ticks. The tick species were identified by comparing important morphological characters such as scutum pattern, spiracle plate, coxa structure, adanal plates, and capitulum shape of different species (26–28).

### 2.4. Data analysis

The prevalence data, including agro-climatic zones, host type, genus-based and host-based infestation of ticks in specific agro climatic zones, were analyzed using Jomovi 1.6.23 software, and R language (version 4.0.5) was used to perform the statistical analysis.

## 3. Results

### 3.1. Tick prevalence on livestock and associated animals

A total of 2,846 animals, of different genders and climatic zones, were selected and investigated for ticks, and 408 animals (66 buffaloes, 106 cattle, 38 sheep, 64 goats, 20 camels, 46 donkeys, 32 horses, and 36 dogs) were found to be infested with ticks. The prevalence of tick infestation among the different host species was 12.2% for sheep, 12.6% for goat, 11.7% for buffalo, 11.7% for cattle, 19.6% for camel, 27.4% for donkey, 23.5% for horse, and 24.3% for dog (Table 1).

**Table 1:** Host wise prevalence of ticks across Pakistan.

**Host**	**Total animals inspected**	**Tick-infested**	**Non-infested**	**Prevalence (%)**
Sheep	312	38	274	12.2
Goat	508	64	444	12.6
Buffalo	564	66	498	11.7
Cattle	908	106	802	11.7
Camel	102	20	82	19.6
Donkey	168	46	122	27.4
Horse	136	32	104	23.5
Dog	148	36	112	24.3

### 3.2. Host-wise prevalence of ticks in different zones

Variations in the tick infestation rate were detected in the different agro-ecological zones of Pakistan. For sheep, the highest prevalence of tick infestation (20%) was observed in zone 5, where the annual precipitation level is moderate to high (200–1,000 mm). For goats, a high percentage of tick infestation (16.7%) was detected in zone 1. The buffaloes in zone 6 showed a tick infestation rate of 15.6%. This zone receives the highest annual precipitation among all 10 zones (1,000–3,000 mm). Cattle showed high tick infestation (13.6%) in zone 4, which is an intermediate precipitation zone. Camels showed the highest infestation rate in zone 1, which is a low precipitation zone. Donkeys showed 39.3 and 33.3% tick infestation rates in zones 4 and 6, respectively. The overall prevalence rate of tick infestation in horses was 25% in zones 2 and 10. The prevalence of tick infestation in dogs was observed to be 31.8 and 30% in zones 4 and 8, respectively. The host-based prevalence of ticks in the agro-ecological zones is provided in Table 2.

**Table 2:** Host based prevalence of ticks in agro-ecological zones of Pakistan.

**Host**	**Animals**	**Zone 1**	**Zone 2**	**Zone 3**	**Zone 4**	**Zone 5**	**Zone 6**	**Zone 7**	**Zone 8**	**Zone 9**	**Zone 10**
Sheep	Total	24	20	48	14	15	45	44	54	28	20
	Infested	2	2	4	2	3	7	4	8	4	2
	**Prevalence**	**8.3%**	**10%**	**8.3%**	**14.3%**	**20.0%**	**15.6%**	**9.1%**	**14.8%**	**14.3%**	**10%**
Goat	Total	24	54	24	54	56	94	74	76	20	32
	Infested	4	6	2	8	8	14	8	6	2	5
	**Prevalence**	**16.7%**	**11.1%**	**8.3%**	**14.8%**	**14.3%**	**14.9%**	**10.8%**	**7.9%**	**10%**	**12.5%**
Buffalo	Total	26	26	96	130	98	90	26	30	28	14
	Infested	2	4	12	14	10	14	4	2	2	2
	**Prevalence**	**7.7%**	**15.4%**	**12.5%**	**10.8%**	**10.2%**	**15.6%**	**15.4%**	**6.7%**	**7.1%**	**14.9%**
Cattle	Total	34	62	116	162	140	100	158	76	30	30
	Infested	4	8	14	22	14	12	16	10	2	4
	**Prevalence**	**11.8%**	**12.9%**	**12.1%**	**13.6%**	**10%**	**12%**	**10.1%**	**13.1%**	**6.7%**	**13.3%**
Camel	Total	28	30	0	0	0	18	0	12	0	14
	Infested	6	8	0	0	0	2	0	2	0	2
	**Prevalence**	**21.4%**	**26.7%**	**0.0%**	**0.0%**	**0.0%**	**11.1%**	**0.0%**	**16.7%**	**0.0%**	**14.9%**
Donkey	Total	0	0	8	56	24	18	28	24	0	10
	Infested	0	0	2	22	4	6	6	4	0	2
	**Prevalence**	**0.0%**	**0.0%**	**25%**	**39.3%**	**16.7%**	**33.3%**	**21.4%**	**16.7%**	**0.0%**	**20%**
Horse	Total	0	8	14	32	28	44	0	2	0	8
	Infested	0	2	2	10	4	10	0	2	0	2
	**Prevalence**	**0.0%**	**25%**	**14.3%**	**31.3%**	**14.3%**	**22.7%**	**0.0%**	**100%**	**0.0%**	**25.0%**
Dog	Total	0	8	22	44	26	14	14	20	0	0
	Infested	0	2	4	14	4	4	2	6	0	0
	**Prevalence**	**0.0%**	**25.0%**	**18.2%**	**31.8%**	**15.4%**	**28.6%**	**14.3%**	**30.0%**	**0.0%**	**0.0%**

Z1 (Hyderabad), Z2 (Dadu, Sukkur, Shikarpur, Rohri), Z3 (Muzaffargarh, Mianwali, Sargodha), Z4 (Vehari, Okara, Sargodha, Mardan, Charsadda), Z5 (D I Khan, Bannu, Mianwali, Chakwal, Attock, Abbotabad, Rawalpindi, Jhelum), Z6 (Rawalpindi, Islamabad, Abbotabad, Swabi), Z7 (Lower Dir, Upper Dir), Z8 (Bannu, Lakki Marwat, Quetta), Z9 (Dadu), Z10 (D I Khan).

### 3.3. Genus-based prevalence of ticks in different zones

The highest infestation rate (71.4%) of *Hyalomma* was observed in zone 1. The most dominant species in zone 1 was *Hyalomma dromedarii*, and the infestation rates of *Hyalomma* in zone 9 and zone 3 were 60 and 55%, respectively. The highest infestation rate of *Rhipicephalus* species (75%) was observed in zone 7, which is located in a high precipitation area. *Haemaphysalis* species showed infestation rates of 40 and 32.4% in zones 9 and 6, respectively. *Ixodes* species showed infestation rates of 26.1 and 25% in zones 8 and 10, respectively (Table 3).

**Table 3:** Genus-based proportion of ticks in agro-ecological zones of Pakistan.

**Tick genus**	**Tick species**	**Zone 1**	**Zone 2**	**Zone 3**	**Zone 4**	**Zone 5**	**Zone 6**	**Zone 7**	**Zone 8**	**Zone 9**	**Zone 10**
*Hyalomma*	*Hyalomma anatolicum*	0	6	14	0	10	0	0	16	6	2
	*Hyalomma scupense*	4	4	4	0	2	0	0	4	0	0
	*Hyalomma dromedarii*	6	4	4	6	0	0	0	2	0	0
	*Hyalomma isaaci*	0	2	0	0	0	0	0	0	0	0
	**Infestation rate**	**71.4%**	**47.1%**	**55.0%**	**6.5%**	**25.0%**	**0.0%**	**0.0%**	**47.8%**	**60.0%**	**12.5%**
*Rhipicephalus*	*Rhipicephalus microplus*	4	8	10	42	12	24	26	8	0	6
	*Rhipicephalus haemaphysaloides*	0	10	0	16	6	10	4	4	0	2
	*Rhipicephalus turanicus*	0	0	0	0	0	2	0	0	0	0
	**Infestation rate**	**28.6%**	**52.9%**	**25.0%**	**63.1%**	**37.5%**	**52.9%**	**75.0%**	**26.1%**	**0.0%**	**50.0%**
*Haemaphysalis*	*Haemaphysalis cornupunctata*	0	0	0	4	0	4	6	0	0	0
	*Haemaphysalis montgomeryi*	0	0	0	2	6	2	2	0	2	0
	*Haemaphysalis bispinosa*	0	0	0	0	4	16	0	0	2	2
	**Infestation rate**	**0.0%**	**0.0%**	**0.0%**	**6.5%**	**20.8%**	**32.4%**	**20.0%**	**0.0%**	**40.0%**	**12.5%**
*Ixodes*	*Ixodes kashmiricus*	0	0	8	22	8	10	2	12	0	4
	**Infestation rate**	**0.0%**	**0.0%**	**20.0%**	**23.9%**	**16.7%**	**14.7%**	**5.0%**	**26.1%**	**0.0%**	**25.0%**
	**Grand total**	**14**	**34**	**40**	**92**	**48**	**68**	**40**	**46**	**10**	**16**

Z1 (Hyderabad), Z2 (Dadu, Sukkur, Shikarpur, Rohri), Z3 (Muzaffargarh, Mianwali, Sargodha), Z4 (Vehari, Okara, Sargodha, Mardan, Charsadda), Z5 (D I Khan, Bannu, Mianwali, Chakwal, Attock, Abbotabad, Rawalpindi, Jhelum), Z6 (Rawalpindi, Islamabad, Abbotabad, Swabi), Z7 (Lower Dir, Upper Dir), Z8 (Bannu, Lakki Marwat, Quetta), Z9 (Dadu), Z10 (D I Khan).

### 3.4. Genus-based infestation rates of ticks in different animal hosts

Out of 2,846 animals, 408 showed positive results for ticks. A wide variation in tick prevalence was observed among the different host species. Out of 11 identified tick species (Table 4), *Hyalomma dromedarii* was the most prevalent in camels (70%). Similarly, the buffaloes were infested with *Rhipicephalus* (69.7%), with *Rhipicephalus microplus* as the most prevalent species. The goats and sheep were mostly infested with *Haemaphysalis* species (46.9 and 42.1%, respectively). *Ixodes* species were more common in the horses and dogs (75 and 60.9%, respectively). Table 4 provides complete details of tick infestation in different animal hosts.

**Table 4:** Genus based proportion of ticks in different hosts.

**Tick genus**	**Tick species**	**Sheep**	**Goat**	**Buffalo**	**Cattle**	**Camel**	**Donkey**	**Horse**	**Dog**
*Hyalomma*	*Hyalomma anatolicum*	14	6	10	28	0	0	0	0
	*Hyalomma scupense*	2	4	6	4	0	0	0	0
	*Hyalomma dromedarii*	0	0	4	6	14	0	0	0
	*Hyalomma isaaci*	2	0	0	0	0	0	0	0
	**Infestation rate**	**47.4%**	**15.6%**	**30.3%**	**35.9%**	**70.0%**	**0.0%**	**0.0%**	**0.0%**
*Rhipicephalus*	*Rhipicephalus microplus*	4	14	44	60	0	6	2	10
	*Rhipicephalus haemaphysaloides*	0	10	2	6	6	12	4	10
	*Rhipicephalus turanicus*	0	0	0	0	0	0	0	2
	**Infestation rate**	**10.5%**	**37.5%**	**69.7%**	**62.3%**	**30.0%**	**39.1%**	**18.8%**	**61.1%**
*Haemaphysalis*	*Haemaphysalis cornupunctata*	4	10	0	0	0	0	0	0
	*Haemaphysalis montgomeryi*	8	6	0	0	0	0	0	0
	*Haemaphysalis bispinosa*	4	14	0	2	0	0	2	0
	**Infestation rate**	**42.1%**	**46.9%**	**0.0%**	**1.9%**	**0.0%**	**0.0%**	**6.3%**	**0.0%**
*Ixodes*	*Ixodes kashmiricus*	0	0	0	0	0	28	24	14
	**Infestation rate**	**0.0%**	**0.0%**	**0.0%**	**0.0%**	**0.0%**	**60.9%**	**75.0%**	**38.9%**
	**Grand total**	**38**	**64**	**66**	**106**	**20**	**46**	**32**	**36**

### 3.5. Number of collected tick species in four seasons

**Figure 2:**
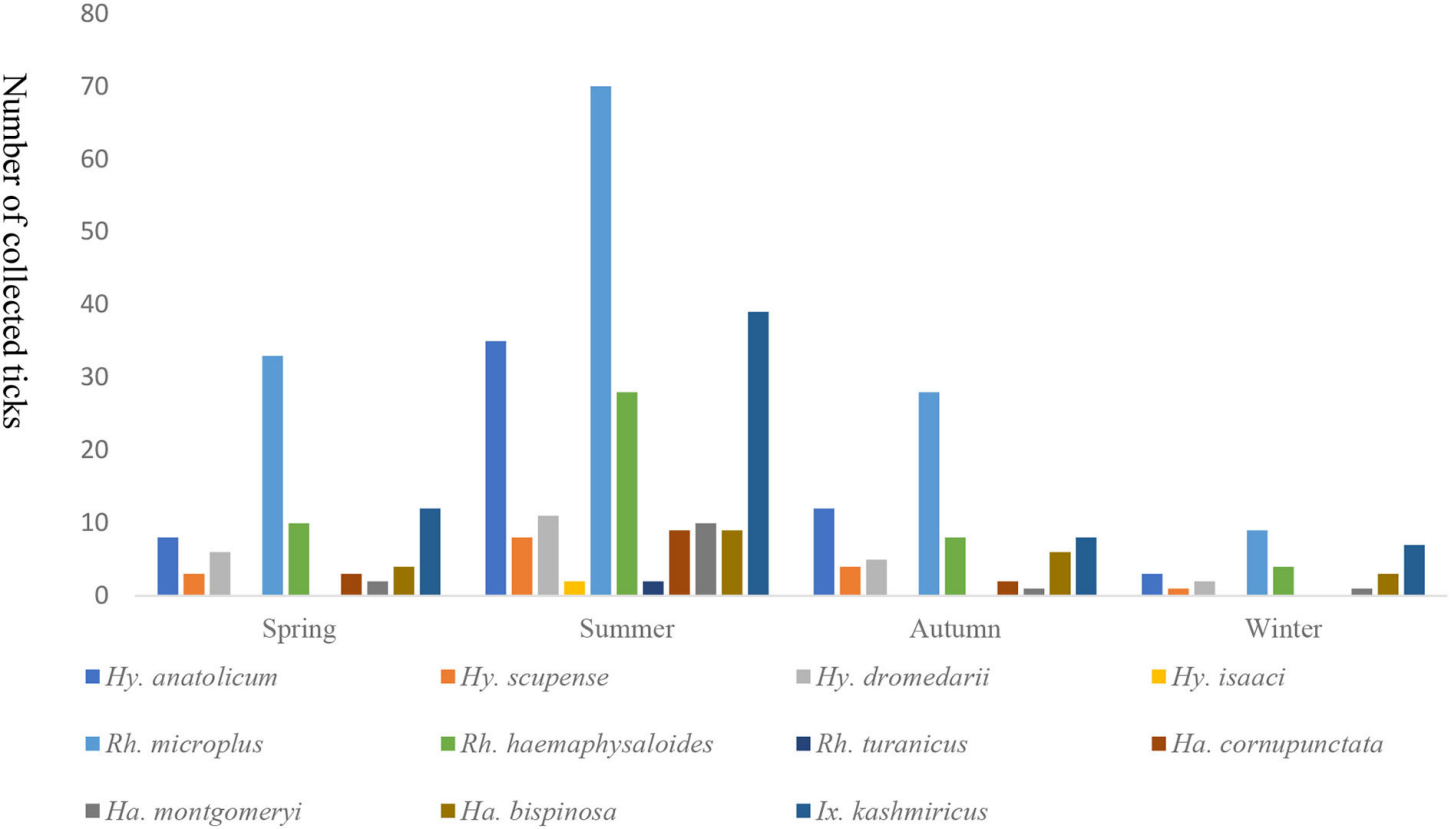
Seasonal prevalence of the reported species in the study region.

Seasonal prevalence showed variations among the four seasons, summer was the most prevalent counting 223 ticks, followed by spring 81, autumn 74 and winter 30 ticks. *Rhipicephalus microplus* was the most common ticks with high prevalence in all seasons, while *Hy. isaaci* and *Rh. turanicus* were the least prevalent ticks among the recorded species, found only in summer season (Figure 2).

## 4. Discussion

In the study area, diverse tick species were observed amongst different livestock and associated animals. The diversity of tick species might be due to the climatic conditions, which are favorable for the development of many tick species (29). The ticks identified in this study are known to be facilitators of several tick-borne infections (30). Several tick species that belong to the genera *Rhipicephalus, Hyalomma, Haemaphysalis*, and *Ixodes* have been reported previously (19, 20, 29–33).

Our findings showed that 11.7% buffaloes, 11.7% cattle, 12.6% goats, 12.2% sheep, 19.6% camels, 27.4% donkeys, 23.5% horses, and 24.3% dogs were infested with ticks. The less infestation rate of ruminants could be due to extra care as they are main source of milk and meat in Pakistan. Moreover, tick infestation rate varied depending on the local climatic and environmental factors, including temperature, humidity, and the presence of host animals (34). Camels showed the highest proportion of tick infestation, which is consistent with the results of previous studies on camels (35). Our study showed that the camels were mostly infested with *Hyalomma* ticks; *Hyalomma dromedarii* showed the highest prevalence (70%), followed by *Rhipicephalus* (30%). Previous studies have shown a similar pattern (35, 36). Camels are mostly found in hot areas in Pakistan. *Hyalomma* highest rate is due top hot and humid climate. Maximum number of eggs of are laid at temperature 34°C and lowest at 15°C and hatched around 32°C and 85% humidity (31). In this study, camels showed the highest prevalence of tick infestation in the hot and dry weather of zone 1. This zone receives an average precipitation of 125–250 mm annually. *Hyalomma* ticks are more resistant to desiccation in general, while some species, including *Hyalomma dromedarii*, are well adapted to survive in dry, even desert, habitats (37). However, few studies have evaluated tick infestation of camels in Pakistan. A previous study performed in Thar Desert, Sindh, reported a tick infestation rate of 80% in camels (35).

To the best of our knowledge, this is the first study of all agro-ecological zones of Pakistan with a wide coverage of livestock as well as livestock-associated equines and canines. Climatic conditions, especially temperature and precipitation, are the most important factors for tick infestation (34, 37, 38). Our study showed a wide variation in tick infestation across the different agro-ecological zones. Sheep showed the highest tick infestation rate (20%) in zone 5. The average annual precipitation in this zone is 200–1,000 mm. Buffaloes showed the highest prevalence of tick infestation in zone 6. The average annual precipitation in this zone is 1,000–3,000 mm. In this study, variations were observed in the prevalence of different tick species according to climatic conditions as well as animal variety (Table 2). We investigated a correlation between agro-climatic zones and tick genera. The infestation rate of *Hyalomma* was the highest in zone 1, which has hot and dry weather conditions; however, the infestation rate of *Rhipicephalus* was the highest in zone 4, which receives moderate precipitation. *Haemaphysali*s ticks were abundant in zone 9. They showed a higher infestation rate in zone 6, which is hot and humid and receives high precipitation; similarly, *Ixodes* showed higher infestation rates in the medium precipitation zones (Table 3). According to previous studies in Pakistan, the tick infestation rate is variable (19, 20, 29–32). High infestation rates of *Rhipicephalus* and *Hyalomma* were reported in Lahore located in zone 4 (32). A previous study in Pakistan covered 5 agro-ecological zones and reported that *Hyalomma* was the most prevalent tick genus in the different zones, followed by *Rhipicephalus* (19). A similar study in 2 districts of lower Punjab in zone 3 reported *Hyalomma* as the predominant genus, followed by *Rhipicephalus* (39). In Multan, sheep and goats showed the prevalence of *Hyalomma* and *Rhipicephalus* infestation (40). A similar study on ticks in Quetta in zone 8 has been reported (41).

The prevalence rate of *Hyalomma* was 70% in camels, followed by sheep and cattle (47.4 and 35.9%, respectively). *Rhipicephalus* was most prevalent genus in buffaloes (69.7%), followed by cattle (62.3%) and dogs (61.1%). *Rhipicephalus* are known as brown dog tick. The highest rate of *Rhipicephalus* is due to presence of dogs and cats near to buffaloes and cows (42). *Haemaphysalis* was more prevalent in goats (46.9%), followed by sheep (42.1%). The infestation rate is almost similar in both ruminants. *Haemaphysalis* is prevalent almost equal in the small ruminants. These results are concordant with the previous studies (43). *Ixodes* is a genus of hard body ticks showed the highest infestation rate in horses (75%), followed by donkeys (60.9%). Highest infestation rate in horses and donkeys is due to landscape and host subject to agriculture, wildlife management and climatic factors (44). Only a few reports are available on horse ticks in Pakistan (8, 9). In this study, we reported *Rhipicephalus turanicus* in dogs. A previous study reported that the prevalence rate of *Rhipicephalus sanguineus* and *Hyalomma anatolicum* in Punjab was about 25% (42, 45). Similar results for *Hyalomma, Rhipicephalus*, and *Haemaphysalis* were reported by a study on livestock in Pakistan (15, 43–48). A previous study reported tick transmission through wild hosts in KPK, Pakistan (15, 49), and a variety of ticks have been detected in small ruminants across Pakistan (47, 50).

## 5. Conclusions

In present study, the prevalence of tick infestation was evaluated in cattle, sheep, goat, buffalo, and camel and their associated equines and canines, such as horse, donkey, and dog, across 10 agro-ecological zones in Pakistan. Eleven tick species from four genera were identified. All of these tick species can transmit a variety of tick-borne pathogens to livestock and humans and pose a substantial threat to the community. The findings of this study will help in the development of locally appropriate tick control strategies based on habitat, animal host, and tick species in Pakistan.

## Data availability statement

The original contributions presented in the study are included in the article/supplementary material, further inquiries can be directed to the corresponding authors.

## Ethics statement

The animal study was reviewed and approved by the Ethical Committee of COMSATS University (under no. CUI/Bio/ERB/2021/42). Written informed consent was obtained from the owners for the participation of their animals in this study.

## Author contributions

NH conducted the study and wrote the manuscript. MA helped in the sampling. AA, JY, RS, and JC revised the manuscript. RS, HA, and JC helped in the research and supervised the study. SU, SI, and SN helped in tick species identification and writing. All authors have read and agreed to the published version of the manuscript.

## Footnotes

^1^ https://timesagriculture.com/agro-ecological-zones-of-pakistan/

^2^ https://en.wikipedia.org/wiki/Animal_husbandry_in_Pakistan

^3^https://www.geo.tv/latest/371999-killing-stray-dogs-should-be-the-last-resort-says-dr-faisal-sultan (2021).

^4^https://www.thenews.com.pk/print/938179-punjab-to-manage-dogs-population (2022).

^5^ https://www.cdc.gov/ticks/.%20October%2021%202021
